# TRPV1 Supports Axogenic Enhanced Excitability in Response to Neurodegenerative Stress

**DOI:** 10.3389/fncel.2020.603419

**Published:** 2021-01-11

**Authors:** Michael L. Risner, Nolan R. McGrady, Andrew M. Boal, Silvia Pasini, David J. Calkins

**Affiliations:** Department of Ophthalmology and Visual Sciences, Vanderbilt Eye Institute, Vanderbilt University Medical Center, Nashville, TN, United States

**Keywords:** TRPV1, neurodegeneration, glaucoma, retinal ganglion cells, axon, dendritic pruning

## Abstract

Early progression in neurodegenerative disease involves challenges to homeostatic processes, including those controlling axonal excitability and dendritic organization. In glaucoma, the leading cause of irreversible blindness, stress from intraocular pressure (IOP) causes degeneration of retinal ganglion cells (RGC) and their axons which comprise the optic nerve. Previously, we discovered that early progression induces axogenic, voltage-gated enhanced excitability of RGCs, even as dendritic complexity in the retina reduces. Here, we investigate a possible contribution of the transient receptor potential vanilloid type 1 (TRPV1) channel to enhanced excitability, given its role in modulating excitation in other neural systems. We find that genetic deletion of *Trpv1* (*Trpv1*^−/−^) influences excitability differently for RGCs firing continuously to light onset (αON-Sustained) vs. light offset (αOFF-Sustained). Deletion drives excitability in opposing directions so that *Trpv1*^−/−^ RGC responses with elevated IOP equalize to that of wild-type (WT) RGCs without elevated IOP. Depolarizing current injections in the absence of light-driven presynaptic excitation to directly modulate voltage-gated channels mirrored these changes, while inhibiting voltage-gated sodium channels and isolating retinal excitatory postsynaptic currents abolished both the differences in light-driven activity between WT and *Trpv1*^−/−^ RGCs and changes in response due to IOP elevation. Together, these results support a voltage-dependent, axogenic influence of *Trpv1*^−/−^ with elevated IOP. Finally, *Trpv1*^−/−^ slowed the loss of dendritic complexity with elevated IOP, opposite its effect on axon degeneration, supporting the idea that axonal and dendritic degeneration follows distinctive programs even at the level of membrane excitability.

## Introduction

Age-related neurodegenerative diseases involve diverse etiologies and phenotypes but share similar patterns of progression (Coleman, 2005; Morfini et al., 2009). Early pathogenesis involves challenges to axonal physiology, including degradation of active transport and accumulation of cytoskeletal components, while somatic and postsynaptic target structures degrade more slowly (Collard et al., 1995; Li and Li, 2004; Stokin et al., 2005; Her and Goldstein, 2008; Crish et al., 2010; Risner et al., 2018). In some instances, early progression includes modified patterns of excitation and excitability. For example, hippocampal brain regions in patients with mild cognitive impairment demonstrate hyperactivation during memory tasks compared to decreased activation in patients with more advanced Alzheimer’s disease (Dickerson et al., 2005), while neurons in transgenic models of the disease show increased action potential generation (Sanchez et al., 2012; Kerrigan et al., 2014; Palop and Mucke, 2016). Similarly, neurons in models of Huntington’s disease and amyotrophic lateral sclerosis demonstrate lower thresholds for spiking accompanied by increased firing and depolarization of the resting membrane potential (RMP; van Zundert et al., 2008; Benraiss et al., 2016; Fogarty, 2018).

Recently, we discovered similar changes in retinal ganglion cell (RGC) excitability in an inducible model of glaucomatous optic neuropathy (or glaucoma), the leading cause of irreversible blindness worldwide (Tham et al., 2014). The disease involves sensitivity to intraocular pressure (IOP), which stresses retinal ganglion cell (RGC) axons where they exit the eye to form the optic nerve (Calkins, 2012). Modest, short-term elevations in IOP transiently enhance RGC excitability, including their light response, even as dendritic arbors lose complexity (Weitlauf et al., 2014; Risner et al., 2018). Enhancement involves IOP-dependent upregulation and translocation of voltage-gated sodium (NaV) channels along the unmyelinated axon segment in the retina, in particular, the Na_V_1.6 subunit (Risner et al., 2018). Interestingly, genetic ablation of the transient receptor potential vanilloid type 1 (TRPV1) channel (*Trpv1*^−/−^) increased Na_V_1.6 and excitability of RGC axons in the optic nerve following elevations in IOP (McGrady et al., 2020), while accelerating axon degeneration (Ward et al., 2014). Finally, elevated IOP changes TRPV1’s net influence on RGC physiology from reducing to promoting excitation (Ward et al., 2014; Weitlauf et al., 2014).

In many neuronal tissues, including the retina, TRPV1 signals stress-related stimuli by modulating excitation through Ca^2+^-driven currents (Rong et al., 2004; Scotland et al., 2004; Jones et al., 2005; Vriens et al., 2009; Sappington et al., 2015). In glaucoma models, RGCs that depolarize to light onset (ON cells) or light offset (OFF cells) may show different susceptibility to elevated IOP (Della Santina et al., 2013; El-Danaf and Huberman, 2015; Ou et al., 2016). Here, we investigate how *Trpv1*^−/−^ influences enhanced excitability of a major class of ON and OFF RGCs, the α-Sustained type (Pang et al., 2003; Della Santina et al., 2013; Ou et al., 2016; Krieger et al., 2017). By comparing wild-type (WT) and *Trpv1*^−/−^ RGC responses to light, we find that TRPV1 has opposing effects on excitability for αON-Sustained (αON-S) and αOFF-Sustained (αOFF-S) RGCs following short-term elevations in IOP. For each type, injecting depolarizing currents to drive voltage-gated channels in conditions that bypass presynaptic activity mimicked the light-induced differences in RGC response. Conversely, we found silencing NaV channels eliminated the differences in the light-evoked activity of WT and *Trpv1*^−/−^ αRGCs, implicating an axogenic source of TRPV1’s influence on RGC physiology. Finally, *Trpv1*^−/−^ slowed the loss of dendritic complexity with elevated IOP, opposite of its effect on axon degeneration (Ward et al., 2014; McGrady et al., 2020). The opposing influence of *Trpv1*^−/−^ on the dendritic organization and axon physiology reinforces the concept that dendritic and axonal degeneration follows distinct programs.

## Materials and Methods

### Animals

Adult male *Trpv1*^−/−^ (B6.129× 1-Trpv1^tm1Jul^/J) mice (1.5–2 months old, *n* = 18) were obtained from The Jackson Laboratory (Bar Harbor, ME, USA), while the appropriate WT background strain C57Bl/6 mice were purchased from Charles River Laboratories (male, 1.5–2 months old, *n* = 18, Wilmington, MA, USA). The *Trpv1*^−/−^ mice have a targeted mutation causing a non-functional truncated form of TRPV1 (Caterina et al., 2000; Ren et al., 2019; Stanford et al., 2019). Mice were maintained in 12 h light/dark cycles, and animals were allowed water and standard rodent chow as desired. All animal experiments were approved by the Vanderbilt University Medical Center Institutional Animal Care and Use Committee.

### Genotyping and Intraocular Pressure Elevation

**Figure 1:**
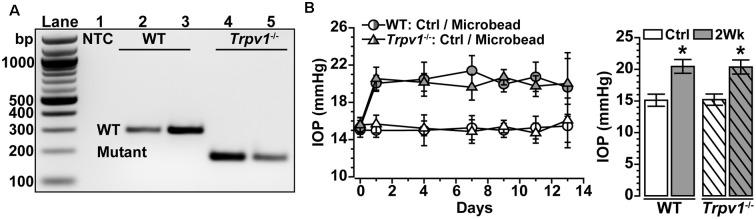
Intraocular pressure (IOP) elevation in *Trpv1^−/−^* mice following microbead occlusion. **(A)** PCR product of wild type (WT) *Trpv1* in C57 mice at 289 bp (lane 2–3) and mutant *Trpv1* at 176 bp (lanes 4–5) compared to no-template control (NTC, lane 1). **(B)** A single unilateral injection of microbeads (1.5 μl) elevates IOP for 2 Weeks in WT (+33%) and *Trpv1*^−/−^ (+36%) eyes compared to an equivalent volume saline injection (Ctrl; *p ≤ 0.003). Post-injection IOP in each eye did not differ between strains (*p* = 0.99; *n* = 18 animals for each. Statistic: one-way ANOVA, Tukey *post hoc* **(B)**. Data = mean ± SEM.

*Trpv1*^−/−^ animals were genotyped before performing experiments, following our protocol (Ward et al., 2014; Weitlauf et al., 2014; Sappington et al., 2015) using primers recommended by the vendor. The mutant forward primer was TAA AGC GCA TGC TCC AGA CT compared to the WT forward primer of TGG CTC ATA TTT GCC TTC AG. The common primer was CAG CCC TAG GAG TTG ATG GA (Integrated DNA Technologies, Coralville, IA, USA). DNA gel electrophoresis of *Trpv1*^−/−^ animals showed a single band at 176 bp indicative of truncated TRPV1 (Caterina et al., 2000; Ren et al., 2019; Stanford et al., 2019), while WT showed a single band at 289 bp indicative of the native protein (Figure 1A). We verified this pattern in each animal utilized. Baseline IOP was measured bilaterally in anesthetized (2.5% isoflurane) mice using TonoPen XL (Reichert Technologies, Depew, NY, USA) for 1–2 days before experimental manipulation. Baseline IOP measurements were averaged (day 0). After baseline IOP measurements, the unilateral elevation of IOP was induced by injecting 1.5 μl of 15 μm polystyrene microbeads (Invitrogen, Carlsbad, CA, USA) into the anterior chamber; the fellow eye received an equal volume of sterile saline to serve as the control. We measured IOP 2–3 times per week for 2 weeks as described previously (Crish et al., 2010; Weitlauf et al., 2014; Risner et al., 2018; Figure 1B).

### Retinal Ganglion Cell Physiology

After euthanizing animals (cervical dislocation), eyes were enucleated, and the retinas were dissected out under long-wavelength illumination (630 nm, 800 μW/cm^2^, FND/FG, Ushio, Cypress, CA, USA). Retinas were placed in carbogen-saturated Ames’ medium (US Biologic, Memphis, TN, USA) supplemented with 20 mM glucose and 22.6 mM NaHCO_3_ (pH 7.4, 290 Osm). Whole retinas were mounted singly onto a physiological chamber and perfused at a rate of 2 ml/min with Ames’ medium maintained at 35°C (Model TC-344C, Warner Instruments, Hamden, CT, USA).

Retinal ganglion cells (RGC) were viewed under DIC using an Andor CCD camera attached to an Olympus BX50 upright microscope at 40×. RGCs were targeted for intracellular recording with pipettes fabricated from borosilicate glass (Sutter Instruments, Novato, CA, USA) and filled with (in mM): 125 K-gluconate, 10 KCl, 10 HEPES, 10 EGTA, 4 Mg-ATP, 1 Na-GTP, and 0.1 ALEXA 555 (Invitrogen, Carlsbad, CA, USA). The intracellular solution pH was 7.35 and osmolarity was 285 Osm. Pipette containing intracellular solution had a resistance between 4–8 MΩ. Whole-cell signals were amplified (Multiclamp 700B, Molecular Devices, San Jose, CA, USA) and digitized at a sampling rate of 50 kHz (Digidata 1550A, Molecular Devices, San Jose, CA, USA). Access resistance was monitored and maintained ≤30 MΩ.

During a typical experiment, we measured RMP, spontaneous spike activity, light-evoked spike activity (full-field 365 nm, 300 μW/cm^2^, 3-s duration, Roithner Lasertechnik, Vienna Austria), and current-evoked spike activity while clamping the cell at 0 pA. In a subset of experiments, we measured light-evoked postsynaptic currents under voltage clamp (−65 mV) while blocking NaV channels with tetrodotoxin (1 μm, TTX). In another subset of experiments, we assessed the influence of TRPV1 antagonism by iodoresiniferatoxin (IRTX, 100 nM, Tocris, Bristol, UK) on light-evoked spiking. After physiology, retinas were placed in 2% paraformaldehyde (PFA) at 4°C for 24 h.

### Immunohistochemistry, Imaging, and Dendritic Morphological Analysis

Following fixation, retinas were immunolabeled for non-phosphorylated neurofilament H (SMI-32, 1:1,000; BioLegend, San Diego, CA, USA) and choline acetyltransferase (ChAT, 1:500; Millipore, Burlington, MA, USA). Retinas were first blocked in 5% normal donkey serum for 2 h and then incubated in primary antibodies for 3 days at 4°C. An Olympus FV1000 inverted microscope was used to obtain micrographs of RGC profiles *en montage*. After imaging, RGC dendritic morphologies were hand traced in Adobe Photoshop, and we measured total dendritic length, number of branching points, and complexity by Sholl analysis (Risner et al., 2018). The total dendritic length was defined as the sum of all dendritic lengths. A branch point was defined as the point of bifurcation of a dendrite from a parent dendrite. We also determined dendritic complexity using Sholl analysis (ImageJ version 1.53c), which measures all dendritic crossings within 10 μm concentric circles from the soma to distal dendritic tips (~300 μm).

### Statistical Analysis

Data are presented as mean ± standard error of the mean (SEM). Graphs were made using Sigma Plot Version 14 (Systat, San Jose, CA, USA) or Graphpad Version 8.0 (Graphpad, San Diego, CA, USA). Statistical analyses were performed using Sigma Plot or Graphpad. Outlier analysis was performed using Grubbs’ test (Graphpad Software, San Diego, CA, USA). After outlier analysis, we determined if data were normal vs. lognormal. If all datasets to be compared best fit a lognormal distribution, data were transformed by taking the logarithm (base 10) of all numbers in the datasets (Choi, 2016). For data that fit a normal distribution or transformed, we performed parametric statistics (Student’s *t*-tests, ANOVAs). Otherwise, we performed non-parametric statistics (Mann–Whitney tests, Kruskal–Wallis tests). Statistical significance was defined as *p* ≤ 0.05.

## Results

### The Influence of *Trpv1*^−/−^ on αON-Sustained RGC Excitability With Elevated IOP

We confirmed the genotype of *Trpv1*^−/−^ mice utilized in these studies by identification of the truncated gene product as described (Figure 1A; Sappington et al., 2009, 2015; Ward et al., 2014; Weitlauf et al., 2014). Daily IOP did not differ between *Trpv1*^−/−^ and WT mice for either control (Ctrl; saline-injected) eyes or eyes receiving a single unilateral injection of polystyrene microbeads for 2 weeks (*p* = 0.99). Microbead injection elevated IOP significantly for both WT (+33%) and *Trpv1*^−/−^ (+36%) eyes compared to their respective control eyes (*p* ≤ 0.003; Figure 1B), consistent with our earlier studies (Ward et al., 2014; Weitlauf et al., 2014). In this study, our WT control group consisted of RGCs from naïve and saline-injected eyes. We did not detect a significant difference in light responses of RGCs from WT naïve vs. saline-injected eyes (*p* ≥ 0.61).

**Figure 2:**
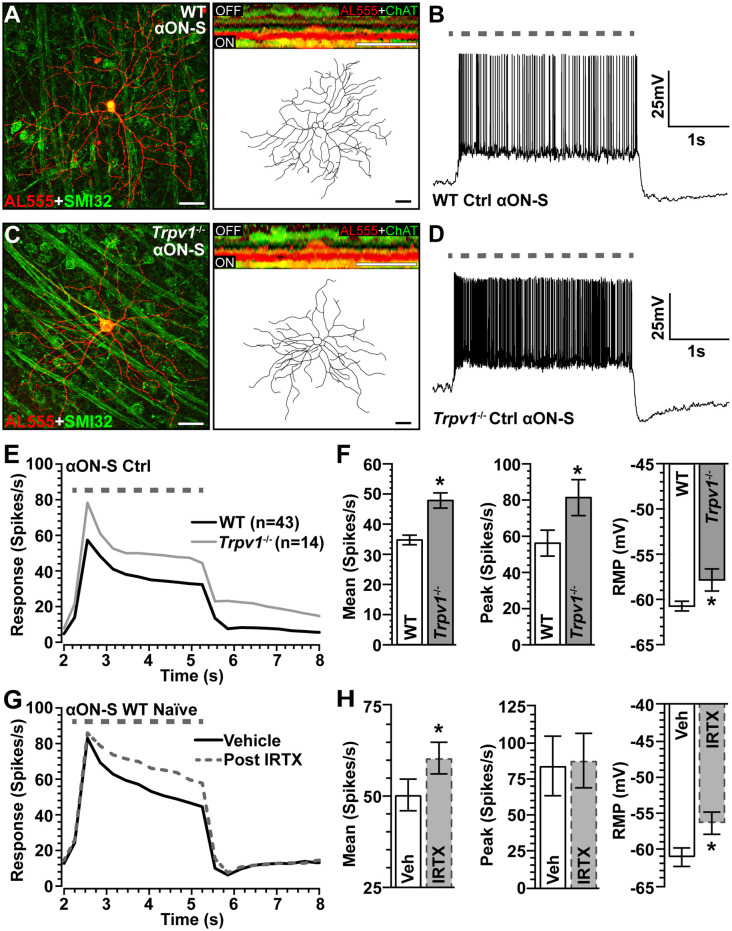
Comparison of WT and *Trpv1*^−/−^ αON-sustained retinal ganglion cells (RGCs). **(A)** Confocal micrograph of WT αON-S RGC following intracellular filling with Alexa555 dye (AL555) shows strong localization of non-phosphorylated neurofilament H (SMI32, green). Orthogonal rotation (inset) shows dendrites ramifying narrowly in the ON region of the inner plexiform layer (IPL) relative to the label for choline acetyltransferase (ChAT, green). **(B)** WT αON-S RGC responds to light (365 nm, 3 s; dashed line) with a sustained train of action potentials during whole-cell current-clamped conditions that preserved resting membrane potential (RMP; 0 pA). **(C)** *Trpv1*^−/−^ αON-S RGC has similar morphology and branching pattern and a more robust sustained response to light **(D)**. When averaged across cells **(E,F)**, the response of *Trpv1*^−/−^ αON-S RGCs exceeded WT for both mean (53.4 ± 7.7 vs. 35.5 ± 4.8 spikes/s; *p < 0.001) and peak firing rate (81.4 ± 9.9 vs. 56.2 ± 7.2 spikes/s; *p = 0.02), with more depolarized (RMP; −57.9 ± 1.1 mV vs. −60.6 ± 0.6 mV, *p = 0.03). **(G,H)** For naïve WT αON-S RGCs (*n* = 7), bath application of IRTX (100 nM) significantly increased the mean light response histogram (+40%; *p < 0.001) and depolarized the RMP (*p = 0.009). Peak response was unaffected (*p* = 0.77). WT control group contains 14 cells from naïve eyes and 29 cells from saline-injected eyes. **(A,C)** Scale bar = 40 μm. Statistics: Student’s *t*-tests **(F)**, paired *t*-tests **(H)**. Data = mean ± SEM.

To assess the impact of *Trpv1*^−/−^ on RGC light responses, we identified αON-Sustained (αON-S) cells using established morphological and physiological criteria (Coombs et al., 2006; Della Santina et al., 2013; Bleckert et al., 2014; Schmidt et al., 2014; Baden et al., 2016; Ou et al., 2016; Risner et al., 2018, 2020). In WT retinas, these RGCs strongly express SMI-32 and have large cell bodies with expansive dendritic fields that ramify narrowly in the ON sublamina of the inner plexiform layer (IPL), proximal to bands of ChAT labeling (Figure 2A). In response to light, these cells produce a sustained train of action potentials (Figure 2B). While αON-S RGCs in *Trpv1*^−/−^ retinas appear smaller, they too strongly express SMI-32 and have similar dendritic morphology and branching (Figure 2C). Their response to light is also sustained though apparently more robust than that of WT αON-S RGCs (Figure 2D). When quantified, the light response of *Trpv1*^−/−^ αON-S RGCs did indeed significantly exceed that of WT, for both mean firing rate (+50%, *p* < 0.001) and peak firing rate (+45%, *p* = 0.02; Figures 2E,F). Also, αON-S RGCs from *Trpv1*^−/−^ retinas demonstrated a significantly more depolarized RMP than their WT counterparts (*p* = 0.03; Figure 2F). The difference in light response between WT and *Trpv1*^−/−^ αON-S RGCs is largely due to the absence of TRPV1 activity rather than a compensatory developmental response to *Trpv1*^−/−^. Bath application of the TRPV1 receptor-specific antagonist iodoresiniferatoxin (IRTX) increased the mean light response of naïve WT RGCs, while also depolarizing the RMP (Figures 2G,H). However, the light-evoked peak firing rate of αON-S RGCs was unaffected by IRTX (Figures 2G,H).

**Figure 3:**
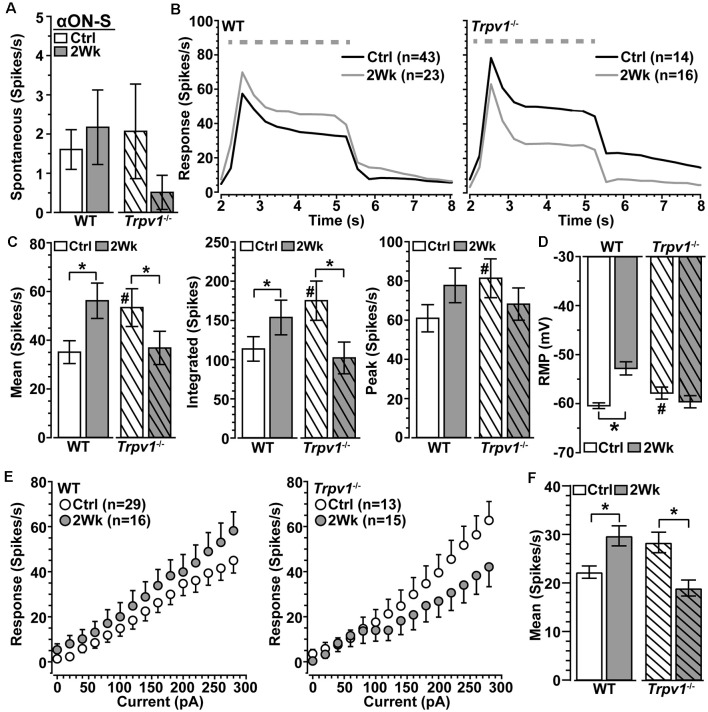
Opposing influence of elevated IOP on WT vs. *Trpv1*^−/−^ αON-S RGCs. **(A)** The spontaneous firing rate of WT and *Trpv1*^−/−^ αON-S RGCs is not affected by elevated IOP (*p* ≥ 0.07). **(B)** Response to light (dashed line) increases for WT αON-S RGCs with elevated IOP but decreases for *Trpv1*^−/−^. **(C)** Elevated IOP increased the mean and integrated light response for WT αON-S RGCs (*p ≤ 0.04), but decreased both for *Trpv1*^−/−^ (*p ≤ 0.05). The integrated response for *Trpv1*^−/−^ Ctrl cells exceeded that for WT (^#^*p* = 0.05), as did mean and peak (#, see Figure 2). **(D)** RMP for WT αON-S RGCS becomes more depolarized with elevated IOP (−53 ± 1.4 vs. −60.6 ± 0.5 mV; *p < 0.001); for control, *Trpv1*^−/−^ was more depolarized (#, see Figure 2). **(E)** The voltage response of WT and *Trpv1*^−/−^ αON-S RGCs following brief (1 s) pulses of depolarizing current (0–280 pA; 2 s inter-stimulus interval). **(F)** Elevated IOP significantly increased voltage response averaged across current pulses for WT αON-S RGCs (*p = 0.01) but decreased it for *Trpv1*^−/−^ compared to Ctrl (*p = 0.002). Statistics: Mann–Whitney test **(A)**, Student’s *t*-tests **(C,D)**, Kruskal–Wallis test, Dunn’s *post hoc* **(F)**. Data = mean ± SEM.

We next asked how *Trpv1*^−/−^ influences excitability for αON-S RGCs stressed by 2 weeks of elevated IOP. Spontaneous activity in the absence of light stimulation was highly variable and did not differ by genotype or IOP (*p* ≥ 0.09; Figure 3A). Elevated IOP had opposing effects on the response to light, increasing it for WT but diminishing it for *Trpv1*^−/−^ αON-S RGCs (Figure 3B). When quantified, elevated IOP increased both the mean response to light for WT (+45%) and the integrated response (+42%), defined by the area under the curve for firing rate during the light stimulus (*p* ≤ 0.04; Figure 3C). The enhanced response is consistent with our previous results (Risner et al., 2018). For *Trpv1*^−/−^ αON-S RGCs, elevated IOP decreased the mean and integrated responses (−38 and −36%, respectively, *p* ≤ 0.05). Interestingly, the enhanced response for WT αON-S cells was approximately equivalent to that of *Trpv1*^−/−^ control cells, while the reduced response for *Trpv1*^−/−^ cells after elevated IOP matched the WT control response (*p* ≥ 0.34). Elevated IOP further depolarized the RMP for WT αON-S RGCs (+14%, *p* < 0.001; Figure 3D), again consistent with our previous finding (Risner et al., 2018), but not for *Trpv1*^−/*-*^cells. To determine if these differences remained in the absence of light-driven presynaptic signaling, we current-clamped αON-S RGCs using incremental (20 pA) injections of depolarizing current to measure intrinsic responses through voltage-gated channels following previous work (Mitra and Miller, 2007). For both WT and *Trpv1*^−/−^ αON-S RGCs, direct depolarization-induced a corresponding increase in firing rate (Figure 3E). However, while elevated IOP enhanced this relationship for WT cells, causing a 33% increase in the mean (*p* = 0.01), we found a 33% decrease for *Trpv1*^−/−^ cells (*p* = 0.002; Figure 3F). Thus, direct activation of voltage-gated channels by depolarizing current steps mirrored the major differences between the light responses of WT and *Trpv1*^−/−^ αON-S cells subjected to elevated IOP. Possible reasons for this effect are discussed below.

### *Trpv1*^−/−^ Has the Opposite Influence on αOFF-Sustained RGC Excitability With Elevated IOP

**Figure 4:**
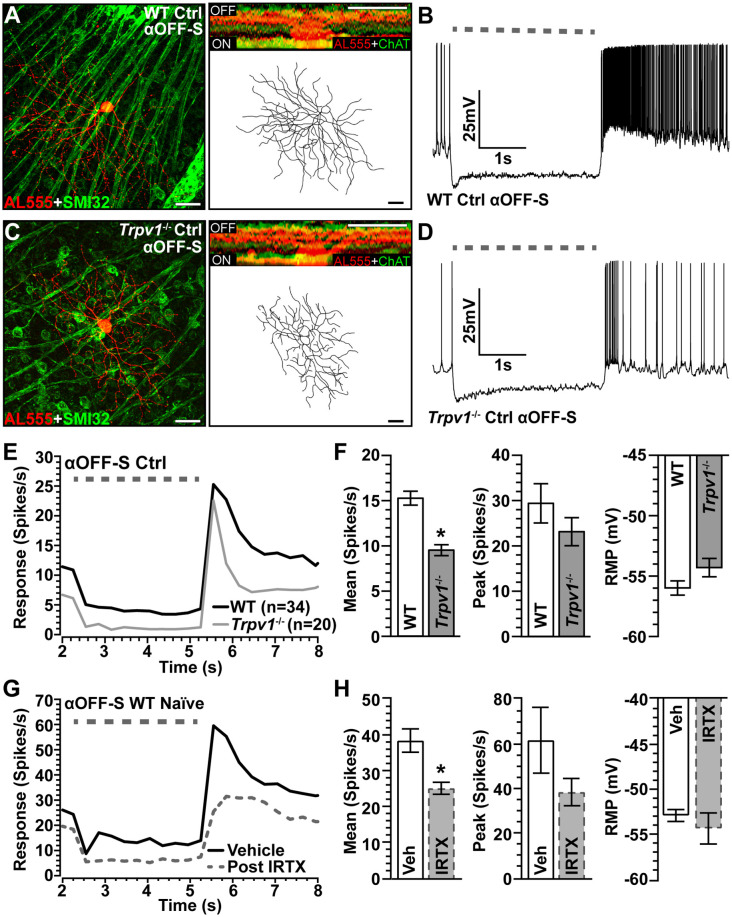
Comparison of WT and *Trpv1*^−/−^ αOFF-sustained RGCs. **(A)** WT αOFF-S RGC following intracellular filling (AL555) and labeled for SMI-32 shows dendrites ramifying narrowly in the OFF region of the IPL proximal to ChAT labeling (inset). **(B)** The voltage response of WT αOFF-S RGC increases and is sustained at the light offset, while excitation diminishes during light stimulation (dashed line). **(C)** *Trpv1*^−/−^ αOFF-S RGC has similar morphology and response to light offset, though less robust than WT **(D)**. When averaged across cells **(E,F)**, the mean response of *Trpv1*^−/−^ αOFF-S RGCs to light offset was less than WT (9.5 ± 0.6 vs. 15.3 ± 0.8 spikes/s; *p < 0.001), though the RMP was slightly more depolarized (−54.3 ± 0.8 vs. −56.0 ± 0.6 mV; *p* = 0.07). **(G,H)** For WT αOFF-S RGCs (*n* = 5), bath application of IRTX (100 nM) reduced the mean response histogram to light offset (−34%; *p < 0.001), though peak off response and RMP were not affected (*p* ≥ 0.14). WT control group consists of 10 cells from naïve eyes and 24 cells from saline-injected eyes. **(A,C)** Scale = 40 μm. Statistics: Mann–Whitney tests **(F)** and paired *t*-tests **(H)**. Data = mean ± SEM.

The mosaic of α-Sustained RGCs contains complementary ON and OFF arrays for partitioning contrast information for use by the visual system (Liang and Freed, 2012). Thus, we identified αOFF-Sustained (αOFF-S) RGCs using analogous criteria (Della Santina et al., 2013; Ou et al., 2016). Like their ON counterparts, WT αOFF-S RGCs have a large cell body with an expansive dendritic field that projects proximal to the complementary ChAT labeling in the OFF sublamina; they too express SMI-32 (Figure 4A). Light suppresses excitation in these cells, which respond with a sustained volley of action potentials at light offset (Figure 4B). The same cell type in *Trpv1*^−/−^ retina had similar morphology but appeared smaller (Figure 4C) with a less robust response to light offset (Figure 4D). When quantified, the *Trpv1*^−/−^ αOFF-S RGC response was significantly less than WT (Figures 4E,F), with a 38% smaller mean response to light offset (*p* < 0.001), though the peak response to light offset did not differ (*p* = 0.96). The RMP for αOFF-S RGCs from *Trpv1*^−/−^ retinas likewise was slightly more depolarized than WT (*p* = 0.07; Figure 4F). As with αON-S RGCs, pharmacological antagonism of TRPV1 with IRTX mimicked the major influence of *Trpv1*^−/−^ on the mean light response of αOFF-S RGCs, causing a decrease (*p* < 0.001; Figures 4G,H).

**Figure 5:**
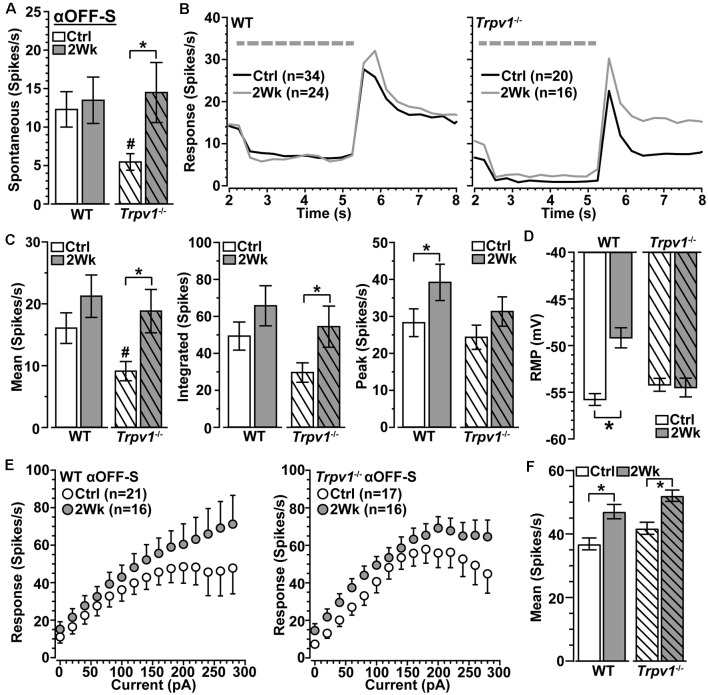
Elevated IOP similarly influences WT and *Trpv1^−/−^* αOFF-S RGCs. **(A)** Spontaneous spike activity of *Trpv1*^−/−^ αOFF-S RGCs is significantly less than WT for control cells (^#^*p* = 0.025) but increased with elevated IOP (*p = 0.022). **(B,C)** Response to light offset increases for both WT and *Trpv1*^−/−^ αOFF-S RGCs, including peak response for WT (^#^*p* = 0.05) and mean and integrated response for *Trpv1*^−/−^ (*p ≤ 0.05); the mean response for *Trpv1*^−/−^ control cells was less than WT (#, see Figure 4). **(D)** Elevated IOP depolarized RMP for WT (*p ≤ 0.001) but not *Trpv1*^−/−^ αOFF-S cells (*p* = 0.81). **(E)** The voltage response of WT and *Trpv1*^−/−^ αOFF-S RGCs following brief (1 s) pulses of depolarizing current (0–280 pA; 2 s inter-stimulus interval). **(F)** Elevated IOP significantly increased response averaged across current pulses for both genotypes compared to respective control cells (*p ≤ 0.002), which did not differ by genotype (*p* = 0.21). Statistics: Student’s *t*-tests **(A,D)**, Student’s *t*-tests (peak response) or Mann–Whitney tests (mean and integrated responses, **C**), Kruskal–Wallis, Dunn’s *post hoc* **(F)**. Data presented as mean ± SEM.

Spontaneous activity in WT αOFF-S RGCs was higher than in αON-S RGCs (see Figure 3A), consistent with their preference for darkness, and did not change with 2 weeks of elevated IOP (*p* = 0.31; Figure 5A). Interestingly, *Trpv1*^−/−^ αOFF-S control RGCs had less spontaneous firing than WT (*p* = 0.025), but this increased with elevated IOP (*p* = 0.05). As expected from our previous work (Risner et al., 2018), elevated IOP enhanced the peak response to light offset for WT αOFF-S RGCs and depolarized the RMP (*p* ≤ 0.04); the mean and integrated responses were modestly enhanced (Figures 5B–D). In contrast to αON-S RGCs, elevated IOP also enhanced the response of *Trpv1*^−/−^ αOFF-S RGCs, increasing both the mean (+87%) and integrated (+84%) response to light offset (*p* ≤ 0.05). Similar to αON-S RGCs, the changes to *Trpv1*^−/−^ αOFF-S RGCs with elevated IOP effectively brought their response to levels of WT control cells (*p* ≥ 0.3). Like *Trpv1*^−/−^ αON-S RGCs, elevated IOP did not change RMP in *Trpv1*^−/−^ αOFF-S RGCs. Once again pulses of depolarizing currents increased the firing rate for all αOFF-S RGCs, though the responses reached a plateau (Figure 5E). Elevated IOP enhanced the mean of current-induced excitability for both WT (+28%) and *Trpv1*^−/−^ (+25%) αOFF-S RGCs compared to their controls (*p* ≤ 0.002; Figure 5F). Thus, as it did for αON-S RGCs, direct activation of voltage-gated channels by depolarizing current steps mirrored the major differences between the light responses of WT and *Trpv1*^−/−^ αOFF-S RGCs.

Our results demonstrate that while elevated IOP enhances excitability in WT αON-S and αOFF-S RGCs, it has an opposing influence on the same cell types in *Trpv1*^−/−^ retina: decreasing excitability for αON-S RGCs while increasing it for αOFF-S cells. These induced changes bring the response of each to that of the corresponding WT control cell.

### An Axogenic Component of TRPV1’s Physiological Influence

Glutamate released from bipolar cell axon terminals conveys light-induced excitation through ionotropic receptors (primarily AMPA-sensitive) localized to postsynaptic sites within RGC dendrites (Wässle, 2004; Euler et al., 2014). Upon binding glutamate, excitation through these receptors enables activation of NaV channels to propagate depolarization in the RGC (Kalbaugh et al., 2009; Sullivan and Miller, 2012). In the retina, NaV subunits localize predominately to RGCs and their axons (Caldwell et al., 2000; Boiko et al., 2003; Werginz et al., 2020), including the Na_V_1.6 subunit that initiates and facilitates the propagation of action potentials and contributes to enhanced excitability (Rush et al., 2005; Hu et al., 2009; Risner et al., 2018). Our results here show that injections of depolarizing current to activate NaV in the absence of conventional light-induced presynaptic excitation captured the major differences in the light-evoked spike rate of WT and *Trpv1*^−/−^ αON-S and αOFF-S RGCs exposed to elevated IOP (Figures 3F, 5F). Next, we conducted the converse experiment to isolate the contribution of conventional excitatory currents to the light response.

**Figure 6:**
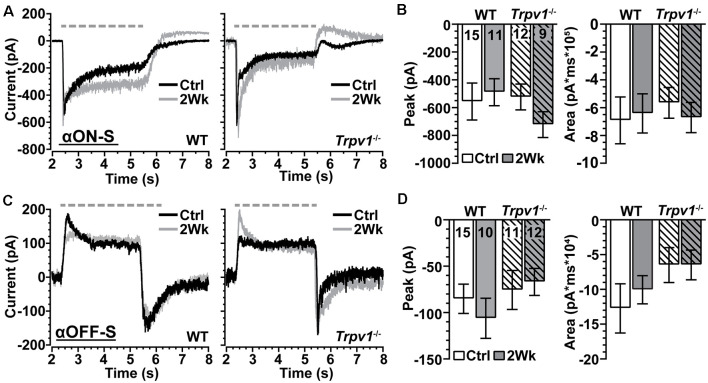
*Trpv1*^−/−^ does not significantly influence excitatory synaptic mechanisms. **(A)** Light-evoked currents (voltage-clamp at −65 mV) of WT and *Trpv1*^−/−^ Ctrl and 2 Week αON-S RGCs following application of tetrodotoxin (TTX, 1 μM). Elevated IOP did not affect the peak (**B**, left) or area of the inward current (**B**, right) for either WT or *Trpv1*^−/−^ 2 Week RGCs compared to their respective Ctrl cells, which also did not differ between WT and *Trpv1*^−/−^ (*p* ≥ 0.31). **(C)** For αOFF-S RGCs, light offset elicited a transient inward peak followed by a slower recovery. Elevated IOP did not affect the peak (**D**, left) or area (**D**, right) of the inward current for either WT or *Trpv1*^−/−^ 2 Week RGCs compared to their respective Ctrl cells, which did not differ between WT and *Trpv1*^−/−^ (*p* ≥ 0.35). Statistics: Kruskal–Wallis tests, Dunn’s *post hoc* tests **(B,D)**. Data = mean ± SEM.

We voltage-clamped WT and *Trpv1*^−/−^ RGCs near the reversal potential of Cl^−^ (−65 mV) and applied tetrodotoxin (TTX, 1 μM) to prevent activation of NaV channels (Pang et al., 2003). Under these conditions, light-induced a transient peak in inward current followed by a sustained component for αON-S RGCs (Figure 6A). For control cells from each genotype, the peak inward current did not differ, and elevated IOP had only a modest effect (*p* = 0.31; Figure 6B, left). The area of the light-evoked inward current also did not change with IOP or differ between WT and *Trpv1*^−/−^ (*p* = 0.88; Figure 6B, right). For WT and *Trpv1*^−/−^ αOFF-S RGCs, light-induced a persistent outward current, while offset elicited a transient inward peak current followed by repolarization (Figure 6C). While repolarization appeared quicker for *Trpv1*^−/−^ αOFF-S RGCs, the peak inward current at light offset was similar to WT for control cells (*p* > 0.99); elevated IOP had no effect on peak current for either WT or *Trpv1*^−/−^ αOFF-S cells (*p* = 0.42; Figure 6D, left). For control αOFF-S RGCs, *Trpv1*^−/−^ reduced the area of the light-evoked inward current, but overall, neither genotype nor IOP elevation affected the area of the inward current (*p* = 0.35; Figure 6D, right). Thus, silencing NaV channels and isolating retinal excitatory signaling eradicated both the differences in light-evoked activity between WT and *Trpv1*^−/−^ RGCs and the changes in response due to elevated IOP.

### *Trpv1*^−/−^ RGC Dendritic Arbors Are Less Susceptible to Pruning

**Figure 7:**
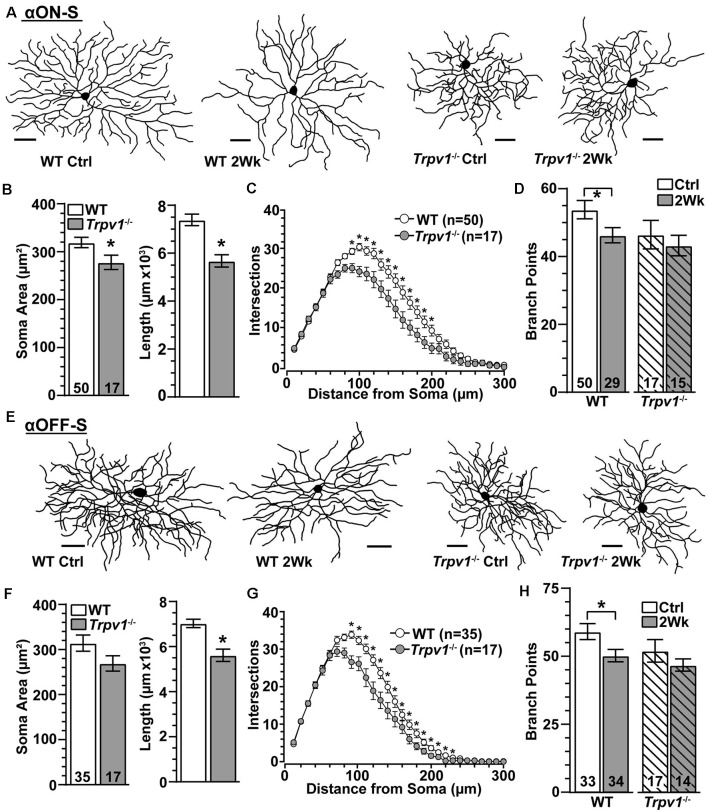
*Trpv1*^−/−^ RGCs are less susceptible to pruning. **(A)** Skeletonized dendritic arbors of αON-S RGCs from *Trpv1*^−/−^ retinas are more compact than WT, which lost branching following 2 weeks of elevated IOP. **(B)** Compared to WT, the mean soma area was smaller (*p = 0.03) and total dendrite length less (*p = 0.002) for *Trpv1*^−/−^ control αON-S RGCs. **(C)** Averaged Sholl analysis for *Trpv1*^−/−^ control αON-S RGCs shows reduced dendritic complexity between 90–200 μm from the soma compared to WT (*p ≤ 0.01). **(D)** Two weeks of elevated IOP reduced the mean number of branch points for WT (*p* = 0.03) but not *Trpv1*^−/−^ αON-S RGCs (*p* = 0.68). **(E)** Dendritic arbors for *Trpv1*^−/−^ αOFF-S RGCs are more compact than WT. **(F)** Compared to WT, total dendrite length was less for *Trpv1*^−/−^ control αOFF-S RGCs (*p < 0.001). **(G)** Sholl analysis for *Trpv1*^−/−^ control αOFF-S RGCs demonstrates reduced dendritic complexity from 90 to 230 μm from the soma (*p ≤ 0.05). **(H)** Elevated IOP again reduced number of dendritic branch points for WT αOFF-S RGCs (*p* = 0.03) but not for *Trpv1*^−/−^ (*p* = 0.48). **(A,E)** Scale = 50 μm. Statistics: Mann–Whitney tests (soma area, **B,F**) and Student’s *t*-tests **(B,D,F,H)**, Two-Way Repeated Measures ANOVA on Ranks, Dunn’s *post hoc* **(C,G)**.

Since *Trpv1*^−/−^ accelerates axon degeneration with elevated IOP (Ward et al., 2014), we asked whether dendritic arbors are similarly susceptible. Two weeks of elevated IOP appeared to reduce branching for WT αON-S RGCs with little or no effect on *Trpv1*^−/−^ cells, which were more compact (Figure 7A), as noted earlier (Figure 2). Indeed *Trpv1*^−/−^ αON-S RGCs from control retina had smaller cross-sectional areas than WT (*p* = 0.045) and less total dendritic length (*p* = 0.002; Figure 7B). Though dendritic arbors for *Trpv1*^−/−^ αON-S RGCs were only 12% smaller in area than WT (*p* = 0.34), they were significantly less complex at a given distance from the soma (Figure 7C). The mean number of branch points per dendritic arbor did not differ from WT (*p* = 0.12). Elevated IOP reduced dendritic branch points for WT (*p* = 0.03) but not *Trpv1*^−/−^ αON-S RGCs (Figure 7D). This brief period of elevation did not affect dendritic field area, total dendritic length, or the number of primary dendrites for either WT (*p* ≥ 0.13) or *Trpv1*^−/−^ αON-S RGCs (*p* ≥ 0.66). We found similar results for αOFF-S RGCs. For *Trpv1*^−/−^ retinas, these cells were more compact (Figure 7E), with significantly less total dendritic length and complexity (Figures 7F,G). Once again, elevated IOP reduced the number of branch points in WT but not *Trpv1*^−/−^ αOFF-S RGCs (*p* = 0.03; Figure 7H), without affecting field area, length, or primary dendrites for either WT (*p* ≥ 0.14) or *Trpv1*^−/−^ αOFF-S RGCs (*p* ≥ 0.45). Thus, α-Sustained RGCs from *Trpv1*^−/−^ retinas are more compact than WT but less susceptible to IOP-related pruning.

## Discussion

### Influence of *Trpv1*^−/−^ on RGC Excitability

Our fundamental finding is that in the absence of induced stress, *Trpv1*^−/−^ increases the light-driven excitability of αON-S RGCs while decreasing the excitability of αOFF-S RGCs (Figures 2, 4). Some 20–40% of all RGCs express TRPV1, as shown with *Trpv1^Cre^*:Ai9 and *Trpv1^Cre^*:AAV^−Flex-tdTomato^ reporter mice (Jo et al., 2017), and about 36% of SMI-32+ αRGCs localize TRPV1, which likely includes αRGCs presented here (Lakk et al., 2018; Figures 2, 4). Retinal horizontal cells also apparently contain TRPV1 (Bouskila et al., 2020). These data are consistent with published accounts of TRPV1 localization in both RGCs and other components of their presynaptic circuitry (Sappington et al., 2009, 2015; Weitlauf et al., 2014). Even so, our data suggest that TRPV1 activation also mediates RGC-intrinsic mechanisms. The polarity of the changes in light response of *Trpv1*^−/−^ RGCs under control conditions can be reproduced by driving membrane potential directly, through injection of depolarizing currents that largely bypass conventional synaptic activity (Figures 3E,F, 5E,F). Additionally, blocking NaV channels and isolating retinal excitatory inputs abolished the differences in light-evoked activity between WT and *Trpv1*^−/−^ control αON-S and αOFF-S RGCs (Figure 6). Thus, while the differential effect of *Trpv1*^−/−^ on αON-S and αOFF-S RGC excitability may reflect differences in TRPV1 expression in either the RGCs or their presynaptic circuits, our results suggest that TRPV1 activation comprises an intrinsic voltage-dependent component.

The impact of *Trpv1*^−/−^ on RGC excitability does not seem to be due to some sort of developmental compensation to change retinal circuitry. This is supported by our finding that antagonism of TRPV1 by IRTX in naïve WT retinas reproduce the changes in light-evoked mean spike rate that we also observed in *Trpv1*^−/−^ αON-S and αOFF-S RGCs (Figures 2E–H, 4E–H). However, we also found *Trpv1*^−/−^ reduces soma area, dendritic length, and complexity of αON-S and αOFF-S RGCs (Figure 7). Since smaller cells have higher input resistance that requires smaller currents to elicit repetitive spiking, RGCs from *Trpv1*^−/−^ retina may be intrinsically more excitable (Kernell, 1966). This possibility is not fully supported by our data. *Trpv1*^−/−^ does not significantly affect the spontaneous firing rate of αON-S cells (Figure 3A), and responses to small test potentials are similar (Figure 3E). Moreover, *Trpv1*^−/−^ αOFF-S RGCs are smaller yet spontaneous activity is reduced (Figure 5A), and responses to low test potentials are like WT control αOFF-S cells (Figure 5E).

Although we found TRPV1 antagonism and *Trpv1*^−/−^ similarly drive light-evoked mean firing in αON-S and αOFF-S cells (Figures 2G,H, 4G,H), *Trpv1*^−/−^ also enhanced peak firing rate of αON-S RGCs; IRTX did not reproduce this change (Figures 2G,H). Since peak firing rate reflects the voltage-gated capacity of a cell, *Trpv1*^−/−^ may induce a compensatory increase in NaV channels that enhances peak spike rate. If so, enhancement of the peak firing rate through NaV channel plasticity likely would require long-term administration of a TRPV1 antagonist. In support of this idea, we have previously found evidence of enhanced localization of Na_V_1.6 in *Trpv1*^−/−^ optic nerve axons (McGrady et al., 2020).

In hippocampal neurons, TRPV1 activation triggers long-term depression, which is reduced by *Trpv1*^−/−^ (Gibson et al., 2008). Similarly, we find *Trpv1*^−/−^ increases excitability and depolarizes RMP of control αON-S (Figures 2E,F). Interestingly, *Trpv1^−/−^* reduces light-driven excitability and modestly depolarizes RMP of control αOFF-S RGCs (Figures 4E,F). On the surface, these findings seem paradoxical. However, αOFF RGCs, unlike αON cells, express low voltage-activated (LVA) Ca^2+^ channels (Margolis and Detwiler, 2007; Margolis et al., 2010). LVA Ca^2+^ channels require hyperpolarization to de-inactivate, rapidly activate to a depolarizing event, and quickly inactivate at low potentials (Margolis et al., 2010). Since *Trpv1*^−/−^ αOFF-S RGCs are intrinsically more depolarized (Figure 4F), the hyperpolarization during light onset may not be strong enough to de-inactivate LVA Ca^2+^ channels, reducing spike output at light offset. When excitability of *Trpv1*^−/−^ control αOFF-S RGCs was measured by depolarizing current injections, spike rate was indistinguishable from WT (Figures 5E,F). These results suggest TRPV1 activity influences multiple voltage-sensitive mechanisms that control RMP and excitability (Khomula et al., 2013; Cazade et al., 2017; McGrady et al., 2020).

### Influence of *Trpv1*^−/−^ on RGC Excitability During Glaucoma

Previously, we found an axogenic mechanism involving increased Na_V_1.6 channels boosts membrane excitability and light responses of αON-S and αOFF-S cells following 2 weeks of elevated IOP (Risner et al., 2018). Once again, we find elevated light responses and increased depolarization of the RMP for WT RGCs following 2 weeks of IOP elevation (Figures 3B–D, 5B–D). With elevated IOP, the *Trpv1*^−/−^ αON-S response to light decreased to the level of WT αON-S RGCs from control retinas (Figures 3B–D), while the *Trpv1*^−/−^ αOFF-S response to light offset increased to the corresponding WT control response (Figures 5B–D). The polarity of the changes in light response with elevated IOP is mirrored by directly activating voltage-gated channels, through injection of depolarizing currents that largely avoid stimulating presynaptic activity (Figures 3E,F, 5E,F). Conversely, blocking NaV channels with TTX and voltage-clamping RGCs to reveal excitatory postsynaptic currents eradicated differences in light-evoked activity caused by IOP elevation for WT and *Trpv1*^−/−^ αON-S and αOFF-S RGCs (Figure 6).

Earlier, we found the net influence of activation of TRPV1 on excitation of RGCs reverses following 2 weeks of elevated IOP, from reducing excitation to increasing; the effect is transient, disappearing by 4 weeks of elevation (Weitlauf et al., 2014). Like its influence on excitability, increased TRPV1 expression in RGCs with elevated IOP is also transient (1–3 weeks; Weitlauf et al., 2014). The reversal in TRPV1’s net influence on excitability with elevated IOP could help explain the opposing effects of *Trpv1*^−/−^ on αRGCs. Following elevated IOP, RMP did not depolarize *Trpv1*^−/−^ αRGCs as it did for WT (Figures 3D, 5D), possibly explaining the higher threshold of depolarization necessary to generate action potentials (Weitlauf et al., 2014). Without TRPV1’s net excitatory influence, *Trpv1*^−/−^ αON-S RGCs demonstrated a lesser response (Figure 3). Following 2 weeks of elevated IOP, despite the absence of TRPV1’s excitatory influence, the *Trpv1*^−/−^ αOFF-S RGC response to light offset increased (Figure 5), just like the response of WT RGCs. This enhanced excitability could be due to differences in Na_V_1.6 expression in *Trpv1*^−/−^ ON and OFF RGC axon initial segments, just as Na_V_1.6 differs between WT and *Trpv1*^−/−^ myelinated axons (McGrady et al., 2020).

Similar to our original report of enhanced excitability (Risner et al., 2018), here we found 2 weeks of IOP elevation reduced the number of dendritic branch points of WT αON-S and αOFF-S RGCs (Figures 7D,H). Surprisingly, IOP elevation did not diminish the dendritic arborization of *Trpv1*^−/−^ RGCs as it did for WT (Figures 7D,H). This finding is perhaps unexpected, given the accelerating influence of *Trpv1*^−/−^ on RGC axon degeneration (Ward et al., 2014). However, TRPV1 activation induces rapid disassembly of dynamic microtubules (Goswami et al., 2006; Weitlauf et al., 2014). With elevated IOP, RGC dendritic arbors demonstrate a certain degree of remodeling as pruning progresses (El-Danaf and Huberman, 2015; Risner et al., 2018), presumably requiring microtubule reorganization and the high degree of energy it requires. With *Trpv1*^−/−^, this process may be hindered, in effect slowing dendritic pruning by preventing the expense of remodeling. The opposing effects of *Trpv1*^−/−^ support the idea that axonal and dendritic degeneration is to some extent independent (Calkins, 2012).

We have suggested that enhanced excitability with elevated IOP may boost RGC signaling to the brain early in glaucoma progression to slow degeneration of RGC axons (Risner et al., 2018). However, *Trpv1*^−/−^ RGCs ultimately fail in this effort, since elevated IOP equalizes the responses of αON-S and αOFF-S RGCs to those of WT control, and *Trpv1*^−/−^ accelerates optic nerve degeneration with elevated IOP (Ward et al., 2014). Even so, enhanced excitability is transient, and RGC axons in WT mice eventually degenerate with sustained IOP elevation. Since the optic nerve is metabolically stressed in glaucoma (Baltan et al., 2010; Calkins, 2012; Coughlin et al., 2015; Inman and Harun-Or-Rashid, 2017; Cooper et al., 2020), the additional burden enhanced excitability places on axons already challenged by limited bioenergetic resources may serve to tip the balance towards degeneration. In this sense, the phenomenon of enhanced excitability could represent both a pro-survival strategy from the standpoint of an individual RGC axon but a pro-degenerative factor for the optic projection.

## Data Availability Statement

The raw data supporting the conclusions of this article will be made available by the authors, without undue reservation.

## Ethics Statement

The animal study was reviewed and approved by the Vanderbilt University Medical Center Institutional Animal Care and Use Committee.

## Author Contributions

MR, NM, and DC designed the research. MR, NM, and SP performed the research. MR, NM, AB, and DC analyzed the data. MR, NM, and DC wrote the aticle. All authors contributed to the article and approved the submitted version.

## Conflict of Interest

The authors declare that the research was conducted in the absence of any commercial or financial relationships that could be construed as a potential conflict of interest.
